# Sex-dependent impairment of antibody responses to tick-borne encephalitis virus vaccination and infection in obese mice

**DOI:** 10.1099/jgv.0.002161

**Published:** 2025-10-06

**Authors:** Michal Dvorak, Dominik Arbon, Jiri Salat, Andrea Fortova, David Pajuelo Reguera, Tereza Frckova, Jiri Holoubek, Jana Balounova, Jan Prochazka, Radislav Sedlacek, Daniel Ruzek

**Affiliations:** 1 Department of Experimental Biology, Faculty of Science, Masaryk University, Brno, Czechia; 2 Veterinary Research Institute, Brno, Czechia; 3 Czech Centre for Phenogenomics, Institute of Molecular Genetics of the Czech Academy of Sciences, Vestec, Czechia; 4 Institute of Parasitology, Biology Centre of the Czech Academy of Sciences, Ceske Budejovice, Czechia

**Keywords:** immune response, obesity, tick-borne encephalitis, tick-borne encephalitis virus, vaccination

## Abstract

*An erratum of this article has been published full details can be found at* *https://doi.org/10.1099/jgv.0.002171*.

Obesity is a growing global health concern with profound effects on immune function and vaccine efficacy. This study investigated the impact of obesity on immune responses to tick-borne encephalitis virus (TBEV) vaccination and infection using a mouse model. Mice on a high-fat diet (HFD) exhibited increased body weight, fat mass and a pre-diabetic state compared to standard chow diet (SCD) controls. After vaccination with the TBEV vaccine (Encepur), HFD mice showed significantly lower TBEV-specific IgG titres and neutralizing antibody levels compared to SCD mice. Splenocyte counts per organ mass were significantly higher in vaccinated SCD mice compared to their HFD counterparts, correlating with the elevated IgG titres observed in the SCD group. These results underscore the critical role of diet in shaping the immune response and vaccine efficacy. Following TBEV infection, HFD mice did not display increased disease severity or elevated viral titres in the serum, spleen or brain relative to SCD controls, indicating that obesity did not exacerbate viral replication or dissemination. However, a sex-dependent effect of obesity on the humoral immune response was observed. Male HFD mice produced antibody titres comparable to their SCD counterparts, suggesting minimal impact of obesity on their immune response. In contrast, female HFD mice exhibited significant impairments in TBEV-specific IgG and neutralizing antibody production compared to female SCD mice, as well as both male HFD and male SCD groups. These findings highlight a complex interplay between obesity, sex and immune function, with obesity disproportionately impairing the immune response after TBEV vaccination and infection.

## Introduction

Obesity has become a global health epidemic, with prevalence rates escalating in recent decades across all age groups and geographic regions [1]. For example, over half of Europeans are overweight or obese [2]. The condition is associated with a wide range of adverse health outcomes, including metabolic disorders, cardiovascular diseases and increased susceptibility to infectious diseases [3,6]. In addition to these well-recognized complications, obesity is increasingly being linked to impaired immune function [7]. Obese individuals often exhibit chronic low-grade inflammation, alterations in cytokine production, changes in immune cell populations and disrupted signalling pathways that collectively contribute to immune dysfunction [7]. These impairments can compromise the body’s ability to respond effectively to infections and reduce the efficacy of vaccines, posing significant challenges to public health, especially in the face of emerging infectious diseases [3].

Tick-borne encephalitis virus (TBEV) is an arthropod-borne orthoflavivirus (*Orthoflavivirus encephalitidis*; genus *Orthoflavivirus*, family *Flaviviridae*) transmitted to humans primarily through the bite of infected Ixodes ticks [8]. Endemic in large parts of Europe and Asia, TBEV causes tick-borne encephalitis (TBE), a severe neurological disease with symptoms ranging from mild febrile illness to debilitating meningoencephalitis and, in some cases, death [8,9]. The incidence of TBE varies significantly between regions, with the highest numbers of reported cases in countries such as the Czech Republic, Germany, Latvia, Lithuania, Russia and Switzerland. Driven in part by global climate change, endemic areas are expanding. Each year, thousands of cases are documented – often peaking during spring and summer when human outdoor activity coincides with tick activity [8,10]. Survivors of TBE often experience long-term neurological sequelae, including cognitive deficits, movement disorders and chronic fatigue [9,11, 12]. Vaccination remains the most effective strategy to prevent TBE, with licensed vaccines providing robust protection in immunocompetent individuals [13]. However, vaccine responses can vary widely among populations, with host factors such as age, sex and pre-existing health conditions playing significant roles in influencing vaccine efficacy [9,13, 14].

Obesity-related immune dysfunction may have critical implications for TBEV immunity. Obese individuals often exhibit impaired humoral responses, reduced vaccine-induced antibody titres and diminished T-cell functionality, which are essential for effective immune defence [3,7]. Moreover, the chronic inflammatory state observed in obesity may interfere with the establishment of long-lasting immunity post-vaccination. In the context of infection, obesity has been associated with delayed pathogen clearance, increased disease severity and heightened risk of complications [3,7].

Numerous studies have demonstrated that obese individuals show reduced antibody responses and impaired maintenance of protective immunity after both natural infection and vaccination. This has been observed for a range of pathogens, including SARS-CoV-2 [15,17], influenza virus [18,21] and hepatitis B virus [22,24]. In the case of arboviruses, several epidemiological studies reported a strong association between obesity and severe infections such as dengue virus, chikungunya virus, West Nile virus, Sindbis virus and Toscana and Sicilian viruses [25,31]. These observations raise the concern that obesity could similarly affect immune responses to TBEV vaccination and infection, potentially compromising vaccine efficacy and exacerbating disease outcomes.

Despite the increasing prevalence of obesity, its impact on TBEV vaccination and infection outcomes has been only minimally investigated. One clinical study examined immune responses following TBEV revaccination in obese individuals compared to normal-weight controls [14]. While TBE booster vaccination was effective in obese individuals, the study revealed a faster decline in antibody levels compared to controls, potentially leading to reduced long-term protection. Obesity also influenced cellular immune responses, as PBMCs from obese vaccinees exhibited elevated interleukin-2 and interferon-*γ* levels upon antigen stimulation. This response suggests a leptin-dependent proinflammatory Th1 polarization, further highlighting the complex effects of obesity on the immune system [14].

In this study, we investigate how obesity influences the immune response to both TBEV vaccination and infection. Using established mouse models, we aim to characterize the effects of obesity on humoral and cellular immunity, vaccine efficacy and disease progression.

By elucidating the interplay between obesity and TBEV immunity, this work seeks to address critical gaps in our understanding of host factors that impair vaccine efficacy and infection control. These findings could inform the development of targeted strategies to improve vaccine responses and clinical outcomes in obese individuals, ultimately contributing to more effective public health interventions for TBE and other infectious diseases.

## Methods

### Virus and cells

TBEV strain Hypr (European subtype, GenBank U39292.1) was passaged five times in the brains of suckling mice and once in BHK-21 cells before being used in this study. This strain was obtained from the Collection of Arboviruses, Biology Center of the Czech Academy of Sciences (https://arboviruscollection.bcco.cz).

Porcine kidney stable (PS) cells were maintained at 37 °C in Leibovitz (L-15) medium with l-glutamine, supplemented with 3% FBS, 100 U ml^−1^ penicillin and 100 µg ml^−1^ streptomycin (Sigma-Aldrich, Czech Republic). The PS cell line was provided by the National Reference Centre for Cell Cultures at the National Institute of Public Health, Prague, Czech Republic [32].

The human lung adenocarcinoma cell line A549 (ATCC CRM-CCL-185) was cultured at 37 °C in a 5% CO₂ environment. Cells were maintained in Dulbecco’s Modified Eagle Medium supplemented with 10% FBS, l-glutamine, 100 U ml^−1^ penicillin and 100 µg ml^−1^ streptomycin.

### Mice and induction of obesity

C57Bl/6NCrl mice (10–12 weeks old for starting the vaccination, 24 weeks old for infection, both sexes) were used in this study. Obesity was induced by feeding mice a high-fat diet (HFD) (D12492, E15742-347, Ssniff, Germany) for ~6 months or until body weight plateaued. Control mice were maintained on a standard chow diet (SCD) (Altromin 1314, Altromin, Lage, Germany) for the same duration.

Health assessments, including body fat composition and intraperitoneal glucose tolerance tests (IpGTT), were conducted at three key time points: before initiating the HFD, during the diet regimen and after the mice reached a stable body weight plateau. Mice were weighed weekly using a precision scale (Radwag PS 3500.X2.M), and their overall health status was closely monitored. Any animals showing signs of distress or suffering were humanely euthanized and excluded from the study.

### Mouse body composition analysis

Body composition, including total body weight, fat mass, lean mass and free fluid mass, was measured non-invasively using time-domain nuclear magnetic resonance (Minispec LF90 II, Bruker, Germany). No anaesthesia or other preparation was required. Each mouse was placed in a plastic cylindrical restrainer (50 mm diameter) designed to limit movement. The restrainer containing the mouse was weighed on a precision scale to determine the total body weight. The restrainer and mouse were then positioned in the Minispec chamber for ~3 min to measure fat mass, lean mass and free fluid mass. After measurement, mice were returned to their original cages.

### Intraperitoneal glucose tolerance test

Mice were subjected to IpGTT following a 16-h fasting period. During fasting, mice were housed in clean cages with unrestricted access to water but no food or faeces. A 20% glucose solution was prepared and administered intraperitoneally at a dose of 2 g of glucose per kilogram of body mass. Blood glucose levels were measured at 0, 15, 30, 60 and 120 min post-injection using a glucometer (FreeStyle Freedom Lite, Abbott, CA, USA). After the procedure, mice were returned to their original cages with their respective diets and access to water.

### Mouse vaccination

Mice were administered subcutaneous injections in the dorsal neck region, with all substances prepared fresh immediately before administration and warmed to room temperature. Each mouse received three doses, spaced 14 days apart, as described previously [33]. The vaccine group received Encepur vaccine (0.25 µg) combined with 10% alhydrogel adjuvant, with a total injection volume of 0.15 ml per dose (15 µl adjuvant in PBS). The Encepur vaccine is based on the inactivated European subtype TBEV, strain K23. It was selected for this study due to its formulation, which excludes human serum albumin as a stabilizing agent, making it more suitable for use in animal models [10,33]. The adjuvant-only group was injected with 10% alhydrogel adjuvant in PBS, also at a total volume of 0.15 ml per dose. The mock group received PBS at the same total injection volume of 0.15 ml per dose. Injections were performed under sterile conditions, and animals were closely monitored post-administration for any adverse reactions or signs of distress. Mice were weighed and anesthetized using isoflurane inhalation anaesthesia. Following anaesthesia, terminal blood collection was performed via the retro-orbital sinus. Blood was allowed to coagulate for 30 min at room temperature before being centrifuged for 10 min at 5,000 g. The resulting serum was carefully collected and immediately frozen at −80 °C for later analysis.

### B-cell subset analysis in the spleen using flow cytometry

Spleens were collected and weighed. Single-cell suspensions were prepared through mechanical dissociation. Upon red blood cell lysis, cells were passed through a 70-µm cell strainer, counted, and 3×10^6^ cells were then stained with a cocktail of fluorescently labelled antibodies targeting B-cell-specific antigens (Table S1, available in the online Supplementary Material 1) supplemented with anti-mouse Fc block (Becton Dickinson 553142) in FACS buffer (2% FCS, 2 mM EDTA, 10 mM HEPES in HBSS w/o Mg2+, Cl2+). After staining, the cells were washed to remove excess antibody and analysed on a Cytek Aurora full-spectrum flow cytometer (5L 16UV-16V-14B-10YG-8R, Cytek Biosciences). Dead cells were excluded based on Hoechst 33258(94403, Merck) fluorescence. Data was analysed in FlowJO software (BD Biosciences). The gating strategy is shown in Fig. S1 in the online Supplementary Material 1.

### Detection of specific anti-TBEV antibodies

The presence of specific anti-TBEV antibodies in mouse sera was assessed using ELISA and virus neutralization tests (VNT).

For ELISA, the IMMUNOZYM FSME (TBE) IgG All Species Kit (Progen, Cat. No. 7701075) was utilized following the manufacturer’s instructions. The concentration of anti-TBEV IgG antibodies was expressed in Vienna units (VIEU/mL).

The VNT was performed as previously described [34]. Briefly, sera from mice were diluted at a 1 : 4 ratio in culture medium, heat-inactivated for 30 min at 56 °C, and serially diluted twofold in a 96-well plate. A 50 µl aliquot of virus stock solution (TBEV strain Hypr, 1×10³ p.f.u. ml^−1^) was added to achieve a final volume of 100 µl. The virus dose was calibrated to produce nearly complete cytopathic effects (CPE), resulting in 90–95% cytolysis. The virus-serum mixture was incubated at 37 °C for 90 min. Subsequently, PS or A549 cells (100 µl, 3×10⁴ cells per well) were added, and the plate was incubated for 4–5 days, allowing visible CPE to develop. The CPE was examined under an inverted microscope (Olympus). Virus neutralization was assessed by determining the endpoint titre, defined as the highest dilution of serum that completely prevented CPE in infected cell cultures, thereby indicating effective viral neutralization.

### Mouse infection

HFD and normal diet mice were subcutaneously infected with TBEV at a dose of 10³ p.f.u. per mouse. Mice were monitored daily for survival, changes in body weight and clinical symptoms, which were scored as follows: 0, no symptoms; 1, ruffled fur; 2, slowing of activity or hunched posture; 3, asthenia or mild paralysis; 4, lethargy, tremor or complete limb paralysis; and 5, death. Mice reaching a clinical score of 4 were humanely euthanized via cervical dislocation upon detection. On days 3 and 7 post-infection (p.i.), three to six mice were euthanized for sample collection. Serum, brain and spleen samples were harvested, processed and analysed for viral titres using plaque assays as described below. Sera were also used for specific anti-TBEV antibody detection as described above.

### Measurement of viral burden

The organs were weighed and homogenized using the Precellys 24 homogenizer (Bertin Technologies), generating 20% (wt/vol) suspensions in L-15 medium supplemented with 3% newborn calf serum. The homogenates were clarified by centrifugation at 5,000×g, and the supernatant was collected for plaque assay. Serum samples were analysed directly without further processing.

To perform the plaque assay, 24-well plates were seeded with PS cells (2.0×10⁵ cells per well) and incubated overnight to establish near-confluent monolayers (~80%). The medium was then replaced with 250 µl of 10×diluted serum or organ homogenate, and the plates were incubated for 4 h at 37 °C. Following incubation, 250 µl of fresh culture medium and 400 µl of 1.5% (wt/vol) carboxymethylcellulose in L-15 medium were added to each well. After 5 days, the plaques were visualized as previously described [34]. Viral titres were calculated and expressed as p.f.u. per millilitre (p.f.u. ml^−1^).

### Statistics

Survival in the mouse experiments was analysed using the log-rank (Mantel–Cox) test in GraphPad Prism 7.04 (GraphPad Software, Inc., USA). Mouse spleen and splenocyte characteristics were evaluated by one-way ANOVA followed by Tukey’s multiple comparisons test using GraphPad Prism 10 (GraphPad Software, Inc., USA). All other data, unless otherwise specified, were analysed using the Mann–Whitney *U* test in GraphPad Prism 7.04. *P*-values<0.05 were considered statistically significant.

## Results

### Diet-induced obese mice exhibit a pre-diabetic state

**Fig. 1.**
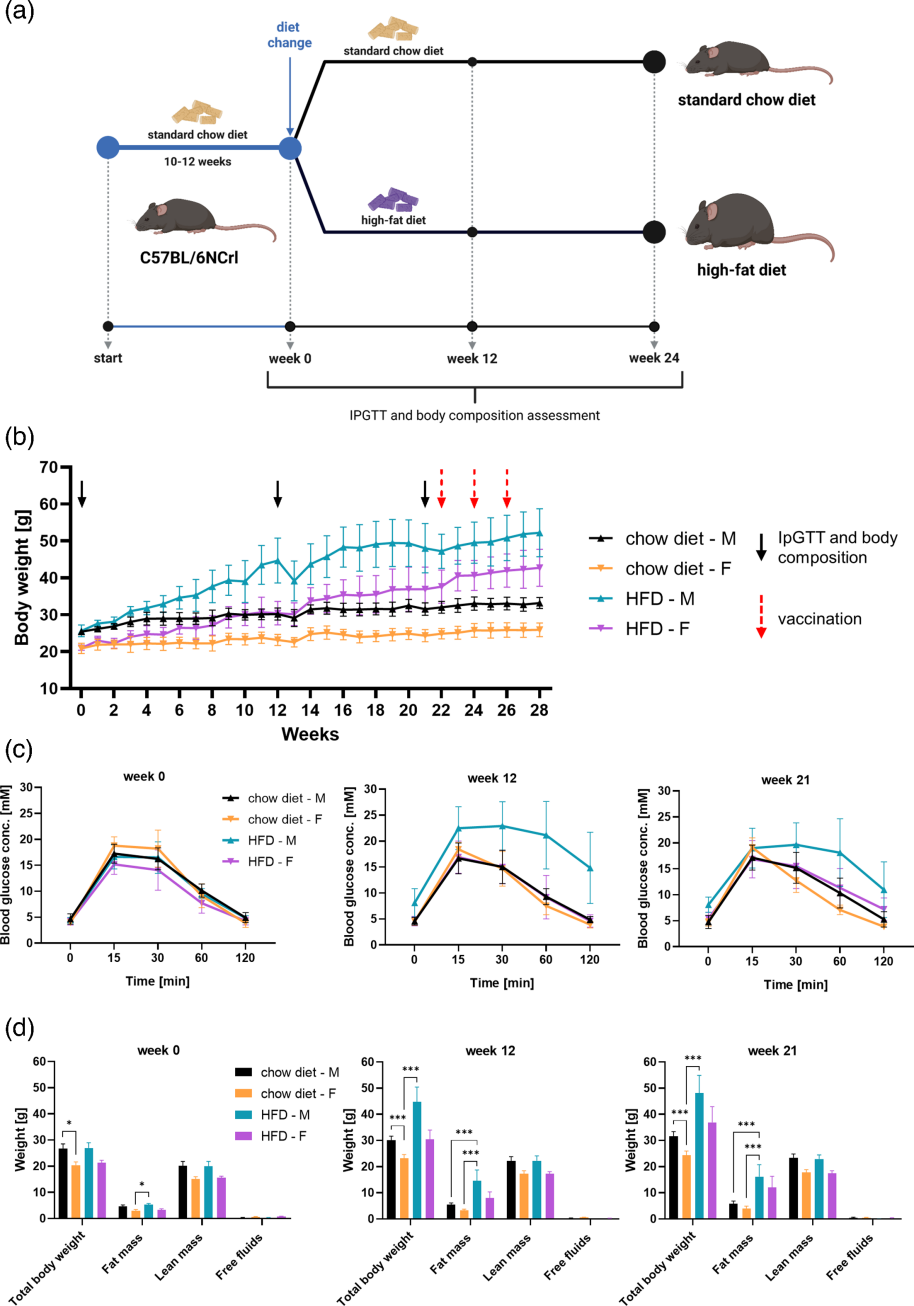
Induction of obesity in mice. (**a**) Study design schematic. Obesity was induced by feeding mice an HFD for ~6 months or until body weight plateaued. Control mice were maintained on a standard chow diet for the same duration. IpGTT and body composition analyses were performed at the indicated time points. (**b**) Body weight curves. HFD feeding resulted in a significant increase in body weight compared to controls. Significant differences at vaccination time points are indicated by red dashed arrows. IpGTT and body composition assessments were performed at time points marked by black arrows. (**c**) IpGTT. This test measured the rate of clearance of intraperitoneally administered glucose. HFD-fed mice displayed prolonged elevated blood glucose levels, indicating a pre-diabetic state, particularly in males. (**d**) Body composition. Significant increases in body weight and fat mass were observed in both sexes in the HFD group compared to controls. Lean mass remained similar, indicating that weight differences were primarily due to increased fat mass. In panels (c–d), the SCD group included 14 males and 14 females, while the HFD group consisted of 11 males and 11 females. *P<0.05; ***P<0.001.

Induction of obesity through an HFD (Fig. 1a) led to a substantial increase in body weight and fat mass, with significant differences observed between the HFD and SCD groups for both sexes (Fig. 1b, d). Lean mass remained unchanged across groups, indicating that the weight differences were primarily attributed to increased fat accumulation due to the HFD. At the initial time point, differences between the groups were minimal, with the only significant variations observed in total body weight for females (*P*=0.033) and fat mass for males (*P*=0.010). However, at subsequent time points, highly significant differences (*P*<0.0005) in total body weight and fat mass were evident for both sexes, further confirming the impact of the HFD on body composition.

IPGTT revealed prolonged elevated blood glucose levels in HFD mice, indicative of impaired glucose clearance and a pre-diabetic state. This effect was particularly pronounced in male HFD mice (Fig. 1c). Lean mass and free fluid content remained consistent across all groups and time points, reinforcing the conclusion that the weight differences observed in HFD mice were exclusively due to an increase in fat mass rather than changes in other body composition parameters.

### Obesity does not affect B-cell populations in the spleen post-vaccination but influences splenocyte counts

**Fig. 2.**
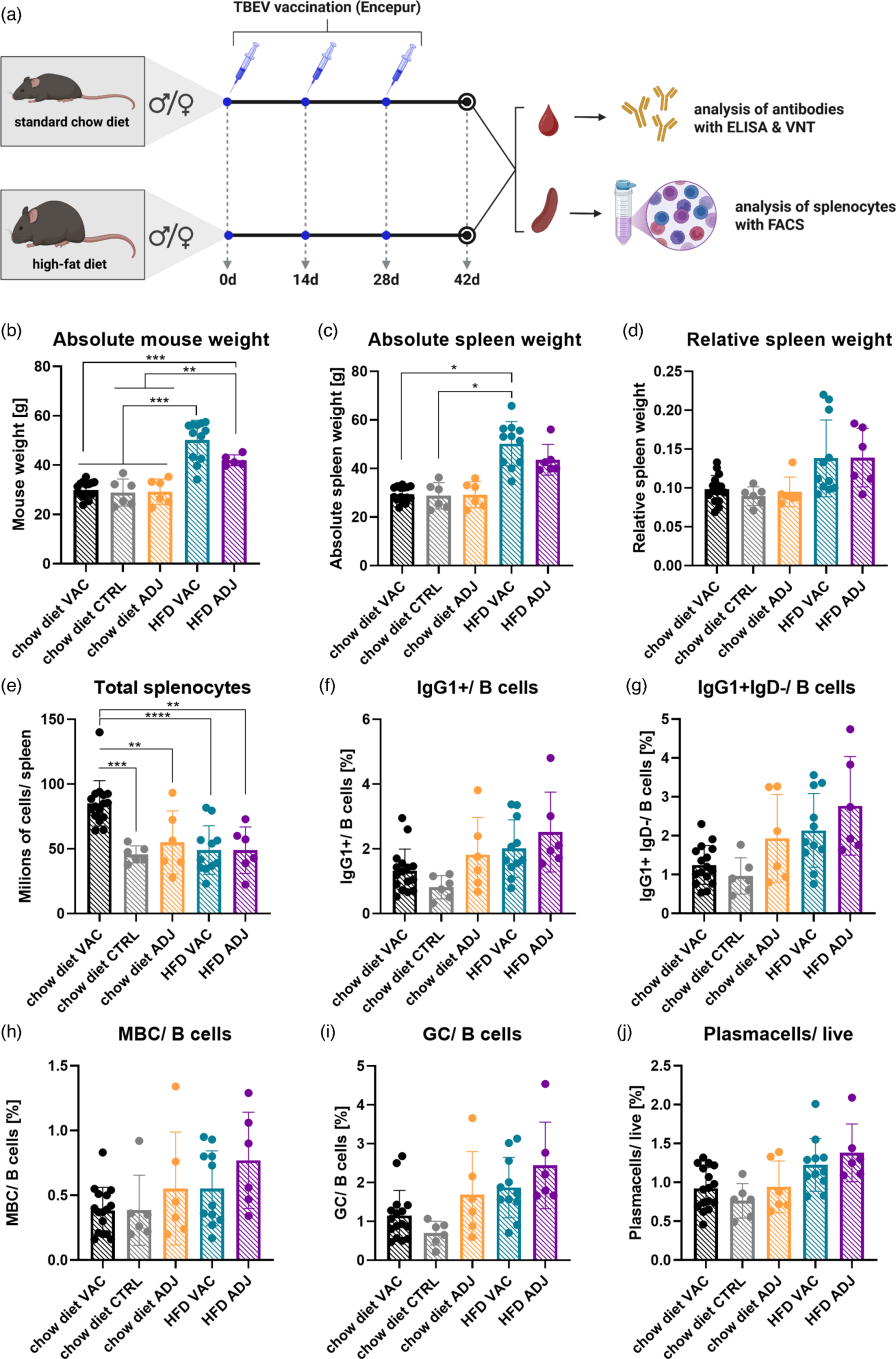
TBEV vaccination in normal and obese mice and effects on spleen and splenocyte characteristics. (**a**) Study design schematic. Mice received three subcutaneous injections of vaccine or adjuvant alone in the dorsal neck region, administered 14 days apart. At the end of the experiment, blood samples and spleens were collected for analysis. (b–d) Spleen characteristics. Obese mice had significantly higher body weight compared to standard chow diet groups (**b**). Absolute spleen weights differed slightly only in vaccinated obese mice (**c**). When spleen weight was expressed relative to body weight, no significant differences were observed between groups (**d**). (**e**) Absolute splenocyte counts. Vaccinated mice on a standard chow diet showed a significant increase in total splenocytes compared to all other groups. (f–j) Analysis of B-cell populations in the spleen by flow cytometry. B-cell populations did not differ significantly between adjuvant and vaccine groups, indicating that vaccination did not alter splenic B-cell composition. The main difference was observed in total splenocyte numbers, with vaccinated mice on a standard chow diet exhibiting a significantly increased count. ADJ, adjuvans only; VAC, TBEV vaccinated; *P<0.05; **P<0.01; ***P<0.001.

Analysis of B-cell populations in the spleens of vaccinated and adjuvant-only mice (Fig. 2a) revealed no significant differences between the groups, indicating that the TBE vaccine did not alter the distribution or ratio of spleen B-cell populations (Fig. 2f–j). Absolute spleen weights showed slight differences, with a marginal increase observed in vaccinated obese mice compared to the other groups (Fig. 2b–d).

In terms of splenocyte counts, vaccinated mice on a SCD exhibited significantly higher absolute splenocyte numbers compared to all other groups, suggesting a distinct response to vaccination in these mice (Fig. 2e) and highlighting a pronounced response to vaccination in the SCD group. This response was absent in both vaccinated and adjuvant-only obese mice, suggesting that the HFD may impair the splenic cellular response to vaccination.

### Obesity impairs antibody responses following TBEV vaccination

To evaluate the effect of obesity on the humoral immune response to TBEV vaccination, HFD mice and control mice maintained on a normal diet were immunized with an inactivated TBEV vaccine. Serum samples were collected to assess both the concentration of TBEV-specific IgG antibodies and the levels of functional TBEV-neutralizing antibodies (Fig. 2a).

**Fig. 3.**
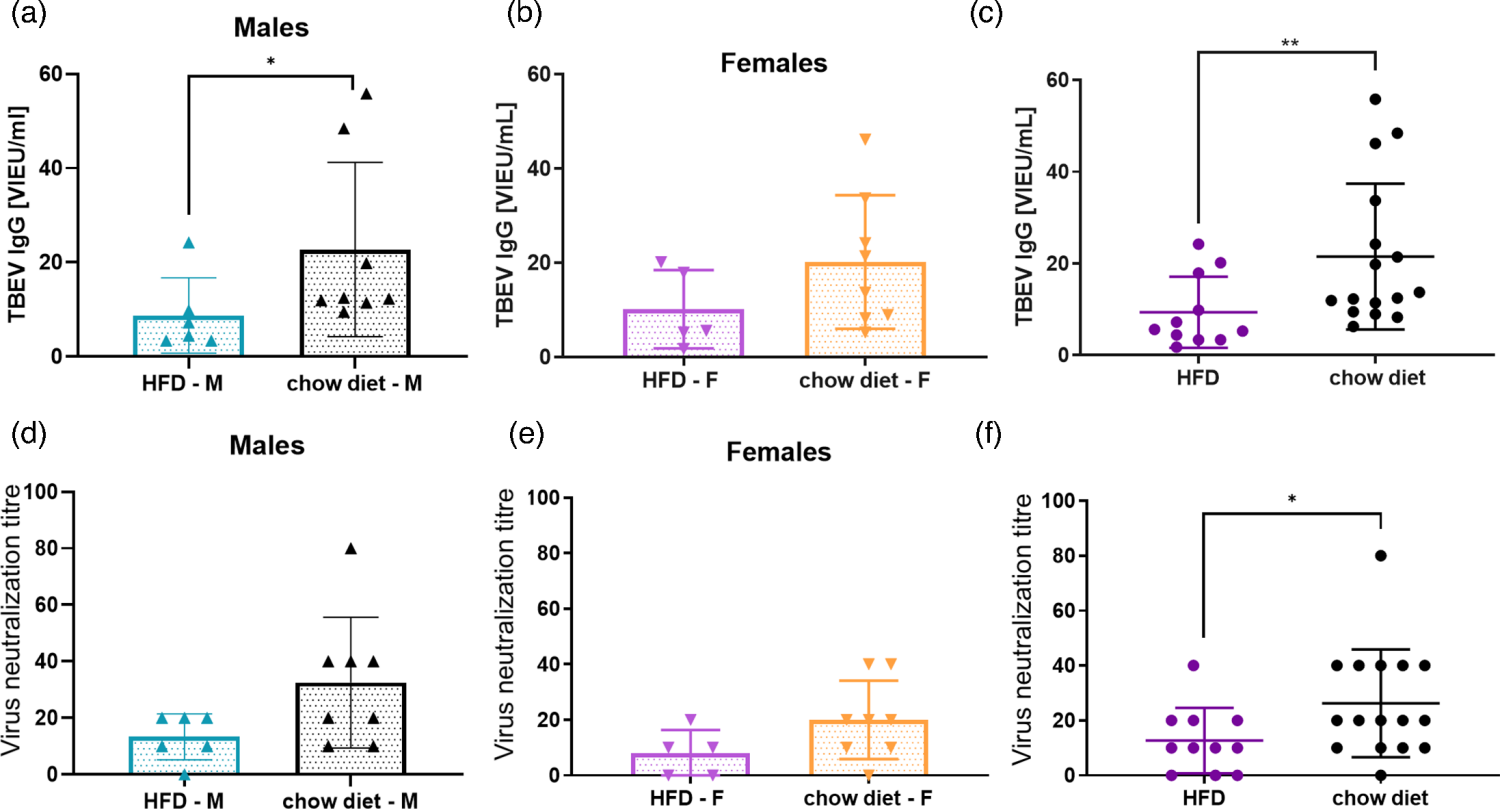
TBEV-specific antibody responses after TBEV vaccination in obese and lean mice. (a–c) TBEV-specific IgG antibodies measured in serum by ELISA. Data are shown separately for males (**a**), females (**b**) and all mice combined (**c**). (d–f) TBEV-neutralizing antibodies measured in serum by neutralization test. Data are shown separately for males (**d**), females (**e**) and all mice combined (**f**). *P<0.05; **P<0.01.

Quantification of virus-specific IgG antibodies by ELISA revealed a significant reduction in antibody titres in HFD mice compared to their lean counterparts. Specifically, control mice exhibited markedly higher IgG levels post-vaccination (*P*<0.01), indicating a diminished antigen-specific humoral response in obese animals (Fig. 3a-c).

This trend was further supported by the results of the virus neutralization assay. Mice on the normal diet developed significantly higher titres of TBEV-neutralizing antibodies than HFD-fed mice (*P*<0.05), suggesting that obesity not only reduces overall antibody production but also impairs the development of functionally protective immunity following vaccination (Fig. 3d–f).

### Obesity does not affect survival time following TBEV infection

**Fig. 4.**
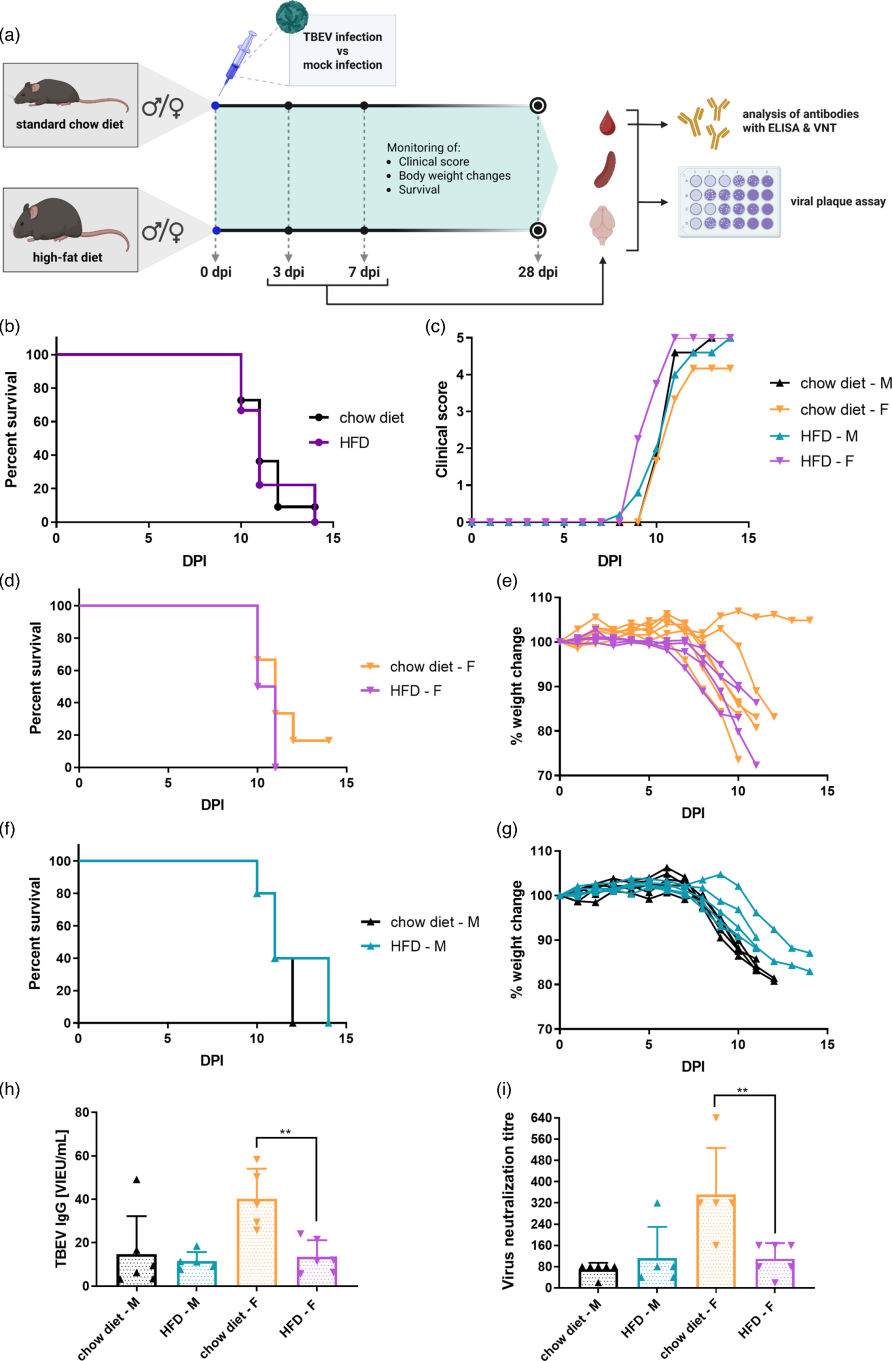
Effect of obesity on TBEV infection in mice. (**a**) Study design schematic. Obese and lean mice were infected with TBEV via the subcutaneous route. Survival, clinical scores and changes in body weight were monitored over 28 days p.i. Organs and blood samples were collected at the indicated time points. (**b, d, f**) Survival curves for all mice combined (*n*=9 HDF mice and 11 chow diet mice) (**b**), females (*n*=4 HFD and six chow diet mice) (**d**), and males (*n*=5 mice per group) (**f**). (**c**) Clinical scores following infection. (**e, g**) Changes in body weight after infection in females (**e**) and males (**g**). (h–i) TBEV-specific antibody responses after infection. (**h**) TBEV-specific IgG antibodies measured in serum by ELISA. (**i**) TBEV-neutralizing antibodies measured in serum by neutralization test. **P<0.01.

To investigate whether obesity influences the course and outcome of TBEV infection, mice of both sexes were fed either an HFD or an SCD and subsequently subcutaneously infected with 10³ p.f.u. of TBEV per mouse. Post-infection, mice were monitored daily for survival, clinical signs and changes in body weight (Fig. 4a).

Survival analysis revealed no statistically significant differences in mean survival time between the HFD and SCD groups in either sex (Fig. 4b, d and f). In all experimental groups, the first deaths or euthanizations due to severe clinical signs occurred around day 10 p.i., followed by rapid mortality in the majority of animals by day 14 p.i., consistent with the known kinetics of TBEV infection in this model. Kaplan–Meier survival curves confirmed that diet-induced obesity did not significantly affect survival probability (log-rank test, *P*>0.05 for all comparisons).

However, subtle differences in disease progression were observed. Female mice on the HFD began to display clinical symptoms, such as ruffled fur, hunched posture and reduced activity, ~1 to 2 days earlier than their SCD counterparts (Fig. 4c). This earlier onset of clinical signs in HFD females was accompanied by a more rapid decline in body weight (Fig. 4e), indicating an earlier onset of systemic illness. Despite this shift in symptom timing, the severity of disease and eventual mortality were similar between HFD and SCD groups, suggesting that obesity may modestly alter the early disease trajectory without affecting ultimate outcomes.

To assess whether obesity affects TBEV replication or dissemination, viral titres were quantified in serum, spleen and brain samples collected from HFD and normal diet mice of both sexes on days 3 and 7 p.i. Infectious viral loads were determined by plaque assay. Overall, no statistically significant differences in TBEV titres were observed between HFD and SCD groups at either time point, regardless of sex (Fig. S2).

On day 3 p.i., TBEV was detectable in the serum of most animals across all groups, indicating systemic dissemination at this early stage. Viral presence in the spleen varied: a higher proportion of HFD female mice had detectable virus (3/6), whereas none of the SCD females showed measurable titres in this organ. In brain samples, a greater proportion of HFD females tested positive for TBEV compared to SCD females (4/6 vs. 1/4), suggesting a potential trend toward earlier neuroinvasion in obese female mice, although this difference was not statistically significant (Fig. S2).

By day 7 p.i., peripheral viral titres declined in most groups, as expected. An exception was observed in SCD females, where four out of five animals still harboured detectable virus in the spleen, in contrast to only one of six HFD mice. Meanwhile, viral titres in the brain increased markedly in all groups by day 7, consistent with central nervous system invasion as the infection progressed. Despite these trends, no consistent pattern of increased or decreased viral burden was found in HFD vs. SCD groups, and intra-group variability remained high (Fig. S2).

### HFD impairs antibody responses to TBEV infection, particularly in female mice

To evaluate whether obesity influences the adaptive immune response following TBEV infection, serum samples collected on day 7 p.i. were analysed for virus-specific IgG and neutralizing antibody levels. TBEV-specific IgG titres were measured using ELISA, while neutralizing antibody activity was assessed by the standard neutralization test.

In male mice, no significant differences were observed in either IgG levels or neutralizing titres between HFD and SCD groups, indicating that diet did not markedly affect the antibody response. In contrast, female mice showed a clear diet-dependent disparity. SCD females mounted significantly higher levels of TBEV-specific IgG compared to SCD males (*P*<0.05), suggesting a sex-dependent enhancement of humoral immunity under normal dietary conditions. However, this robust antibody response was markedly diminished in HFD females, who exhibited significantly lower IgG levels than their SCD counterparts (*P*<0.01) (Fig. 4h).

A similar pattern was observed for TBEV-neutralizing antibody titres. SCD females developed significantly stronger neutralizing responses than SCD males (*P*<0.01), consistent with their higher TBEV-specific IgG levels. Conversely, HFD females showed a significant reduction in neutralizing antibody titres compared to SCD females (*P*<0.01), indicating that obesity impairs the development of functional, virus-neutralizing antibodies (Fig. 4i).

## Discussion

This study investigated how diet-induced obesity influences immune responses to TBEV vaccination and infection, using a well-established mouse model [20,35, 36]. Obesity is increasingly recognized as a key factor that impairs immune function, particularly in the context of viral infections and vaccination efficacy [3,7].

In the case of TBE, clinical observations suggest that obesity may similarly compromise vaccine-induced immunity. Garner-Spitzer *et al.* [14] reported that vaccinated obese individuals showed a more rapid decline in TBEV-specific antibodies, potentially undermining long-term protection, particularly among men [14]. However, controlled experimental evidence directly linking obesity to altered immune responses or disease outcomes in TBEV infection has been lacking. To address this gap, we employed a controlled mouse model to systematically examine the effects of obesity on both vaccine-induced and infection-induced immune responses, with attention to possible sex-specific effects.

Consistent with other models of diet-induced obesity [20,35, 37, 38], mice fed an HFD developed significant increases in body weight and fat mass without changes in lean mass or fluid content, confirming the selective accumulation of adipose tissue. HFD mice also exhibited impaired glucose tolerance, indicative of a pre-diabetic state. These metabolic alterations are known to be associated with chronic low-grade inflammation and immune dysregulation, making this a relevant model to study obesity-associated immune impairment [20,35, 36].

Survival analysis following TBEV infection revealed no significant differences between HFD and SCD groups, indicating that obesity alone does not substantially influence mortality in this model. All groups showed similar survival curves, with mortality initiating around day 10 and peaking by day 14 p.i. – findings consistent with previous studies using lethal TBEV strains [39]. Nevertheless, clinical observations suggest subtle differences: obese female mice exhibited an earlier onset of symptoms and more rapid body weight loss compared to SCD females, hinting at accelerated disease progression in this group.

To explore potential virological correlates of this clinical pattern, we analysed viral loads in serum, spleen and brain at early (day 3) and later (day 7) time points. Although overall viral titres were broadly similar between dietary groups, certain trends emerged. Notably, a higher proportion of HFD females had detectable virus in the brain on day 3, suggesting earlier neuroinvasion in obese mice. By day 7, viral loads in the brain had increased across all groups, consistent with TBEV’s neurotropic nature. While these observations did not reach statistical significance, they imply that obesity may subtly alter early virus dissemination, particularly to the central nervous system, in a sex-dependent manner. Similarly, obesity did not consistently affect viral replication in the blood of animals infected with arthritogenic alphaviruses, except for reduced viral loads in obese mice at later stages of infection. Nevertheless, morbidity was increased in obese mice [40]. Interestingly, sex-associated differences in disease severity and metabolic reprogramming in human TBEV infections have also been reported [41].

Our results are similar in some aspects to findings from other orthoflavivirus models. Specifically, Geerling *et al.* [42] demonstrated that in a mouse model of West Nile virus infection, obese female mice experienced higher mortality, elevated viral titres in the brain and impaired neutralizing antibody responses [42]. Like our study, theirs highlighted the interaction between sex and obesity in shaping immune responses and disease severity in orthoflaviviral infections. Following dengue virus infection, obese mice exhibited more severe morbidity – including greater weight loss and thrombocytopenia – compared to healthy-weight controls. Obesity was also associated with altered cytokine expression [43].

The most striking effects of obesity in our study emerged in the context of the humoral immune response. Obese mice exhibited significantly reduced levels of TBEV-specific IgG and neutralizing antibodies compared to their SCD counterparts, aligning with previous findings reported for West Nile virus infection [42]. This effect was observed in vaccinated mice of both sexes and in infected females. After West Nile virus infection, male mice maintained relatively robust antibody responses regardless of diet, but the antibody production in SCD females was strong and significantly impaired by obesity. These results suggest a female-specific sensitivity of the humoral immune system to obesity-associated dysregulation in West Nile infection [42].

One potential explanation for the observed female-specific effects is the influence of sex hormones on immune responses. Oestrogen has been reported to enhance B-cell function and antibody production, while obesity is associated with disrupted endocrine signalling and hormonal imbalances. It is, therefore, plausible that obese female mice, who may normally benefit from oestrogen-mediated immune advantages, experience greater immune impairment due to altered hormonal regulation [44]. However, this remains a hypothesis and was not directly tested in the present study.

In further support of the hypothesis that obesity impairs the immune response to vaccination, splenocyte analyses in vaccinated mice showed that although the frequency of B cells in the spleen was not significantly different between groups, HFD mice had significantly lower total splenocyte counts and reduced cell density per spleen weight. These findings may indicate a possible disruption in B-cell expansion in secondary lymphoid organs, which could be associated with the weakened humoral responses observed.

Taken together, our data suggest that while obesity does not affect survival after TBEV infection in our experimental model, it is associated with reduced generation of protective antibodies. The fact that these effects were most pronounced in females raises important considerations for sex-specific vaccine strategies and immune monitoring in at-risk populations. The absence of differences in survival times, despite clear variations in symptom onset and antibody responses, is unexpected. One possible explanation is the use of a highly pathogenic TBEV strain, which may have overwhelmed more subtle immunological or clinical differences between groups, thereby masking potential effects on disease progression.

It should also be noted that levels of neutralizing antibodies are not the sole determinants of disease severity following TBEV infection. While they play a critical role in viral clearance and protection, several other host factors can contribute to disease outcome. These include cellular immune responses, such as T-cell activation and cytokine production [41,45, 46], innate immune mechanisms [47,48], genetic background [49,51], age and others. The interplay between these components may significantly affect viral dissemination, neuroinvasion and overall clinical progression. Further investigation into these parameters will be essential to fully understand the immunopathogenesis of TBEV and will be a focus of future studies. The use of a mouse TBE model may also limit the study, as results may not directly apply to humans [52].

This study has several limitations. First, it employed a single TBEV strain. Using a less virulent strain could potentially reveal additional effects, particularly in terms of mean survival times and viral titres across organs. Second, increasing the number of animals in the individual sex groups may enhance the statistical power of some observations. Third, assessing antibody responses at multiple time points post-vaccination would provide a more detailed understanding of the kinetics and durability of the humoral immune response. Finally, while antibody levels are a key correlate of protection, they do not represent the only determinant of disease severity following TBEV infection. Future studies will aim to investigate additional immune parameters (such as cytokine profiling and analysis of T-cell activation) to better characterize the mechanisms underlying the observed effects.

In conclusion, our study highlights the complex interplay between metabolic status, sex and immune function in the context of orthoflavivirus infection. Obesity impairs vaccine- and infection-induced antibody responses to TBEV, with the effect on infection-induced responses being most pronounced in female mice, without significantly impacting overall survival. These findings underscore the importance of considering both obesity and sex as important biological variables in vaccine evaluation and infectious disease research. Further studies are warranted to elucidate the mechanistic basis of this immune impairment and to determine whether similar patterns occur in human populations.

## Supplementary material

10.1099/jgv.0.002161 Supplementary Material 1.

## Abbreviations

CPE: complete cytopathic effects
HFD: high-fat diet
IpGTT: intraperitoneal glucose tolerance tests
p.i.: post-infection
PS: porcine kidney stable
SCD: standard chow diet
TBE: tick-borne encephalitis
TBEV: tick-borne encephalitis virus
VNT: virus neutralization tests
