# Erratum: Sex-dependent impairment of antibody responses to tick-borne encephalitis virus vaccination and infection in obese mice

**DOI:** 10.1099/jgv.0.002171

**Published:** 2025-10-29

**Authors:** Michal Dvorak, Dominik Arbon, Jiri Salat, Andrea Fortova, David Pajuelo Reguera, Tereza Frckova, Jiri Holoubek, Jana Balounova, Jan Prochazka, Radislav Sedlacek, Daniel Ruzek

**Affiliations:** 1 Department of Experimental Biology, Faculty of Science, Masaryk University, Brno, Czechia; 2 Veterinary Research Institute, Brno, Czechia; 3 Czech Centre for Phenogenomics, Institute of Molecular Genetics of the Czech Academy of Sciences, Vestec, Czechia; 4 Institute of Parasitology, Biology Centre of the Czech Academy of Sciences, Ceske Budejovice, Czechia

In the original version of this article, a production error led to the term titres being replaced by litres throughout the article. This distorted the meaning of the text, and as such, the version of record has been updated to rectify the error.

The Microbiology Society apologises for any inconvenience caused.

