# Supplementary material for: Sex-dependent impairment of antibody responses to tick-borne encephalitis virus vaccination and infection in obese mice
Source: J Gen Virol. 2025 Oct 6;106(10):002161. doi: 10.1099/jgv.0.002161 (PMC12500394; doi:10.1099/jgv.0.002161)
Supplement: Supplementary Material 1. [file jgv-106-02161-s001.pdf]

Supplementary data

Sex-Dependent Impairment of Antibody Responses to Tick-Borne Encephalitis Virus Vaccination and Infection in Obese Mice

Michal Dvorak,[1,2#] Dominik Arbon,[3#] Jiri Salat,[1,2,4#] Andrea Fortova, [1,2,4] David Pajuelo Reguera,[3] Tereza Frckova,[1,4] Jiri Holoubek, [1,2,4] Jana Balounova,[3] Jan Prochazka,[3] Radislav Sedlacek,[3] Daniel Ruzek[1,2,4,✉]

(1) Department of Experimental Biology, Faculty of Science, Masaryk University, Brno, Czechia

(2) Veterinary Research Institute, Brno, Czechia

(3) Czech Centre for Phenogenomics, Institute of Molecular Genetics of the Czech Academy of Sciences, Vestec, Czechia

(4) Institute of Parasitology, Biology Centre of the Czech Academy of Sciences, Ceske Budejovice, Czechia

# Equal contribution

✉ Author for Correspondence: ruzekd@paru.cas.cz; ruzek@sci.muni.cz

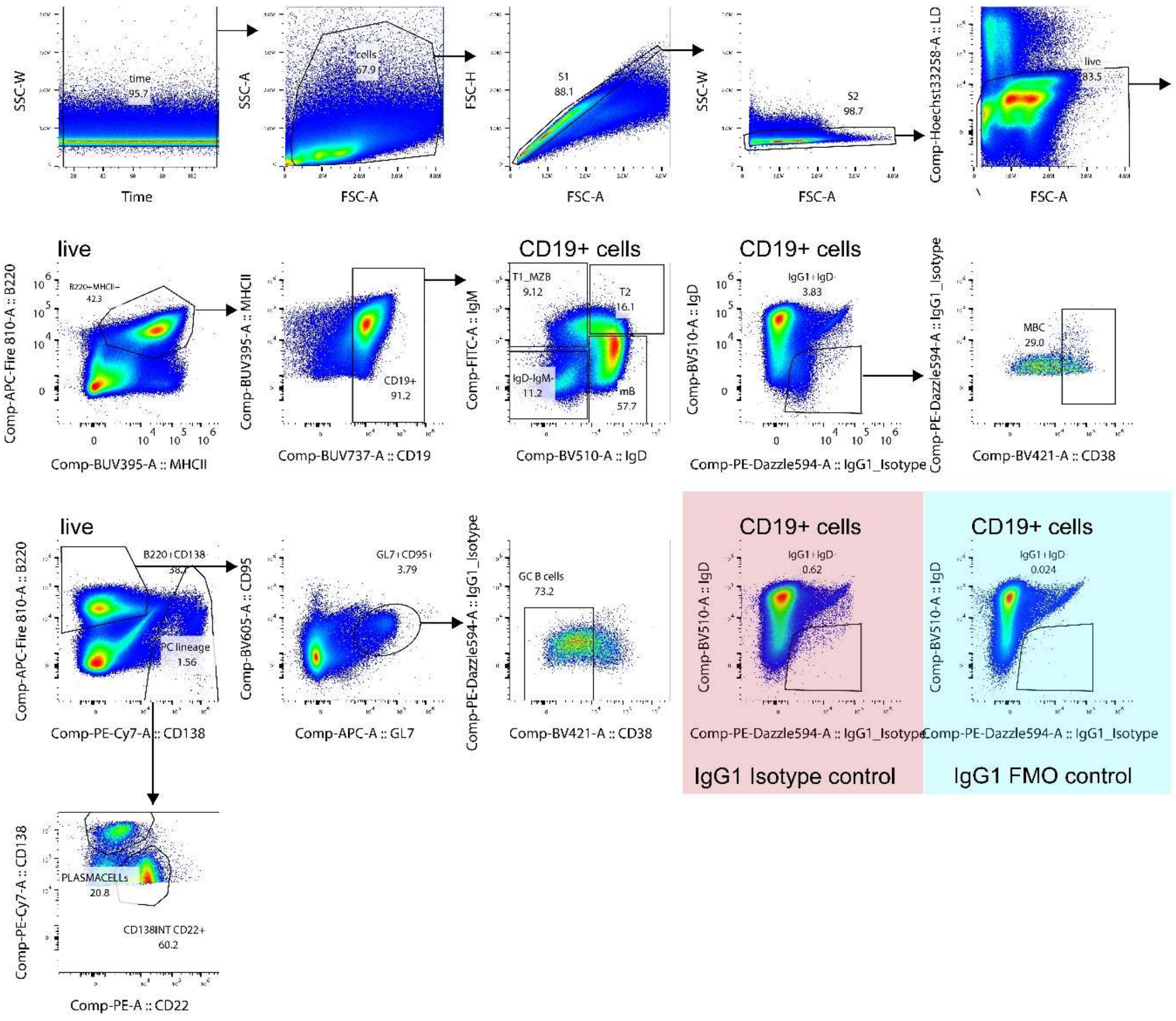

Supplementary Figure 1 | Gating strategy for flow cytometry analysis. A spleen sample from a female, HFD, adjuvant, was used. Clearly defined gated populations are visible. Corresponding isotype and FMO controls are included for comparison.

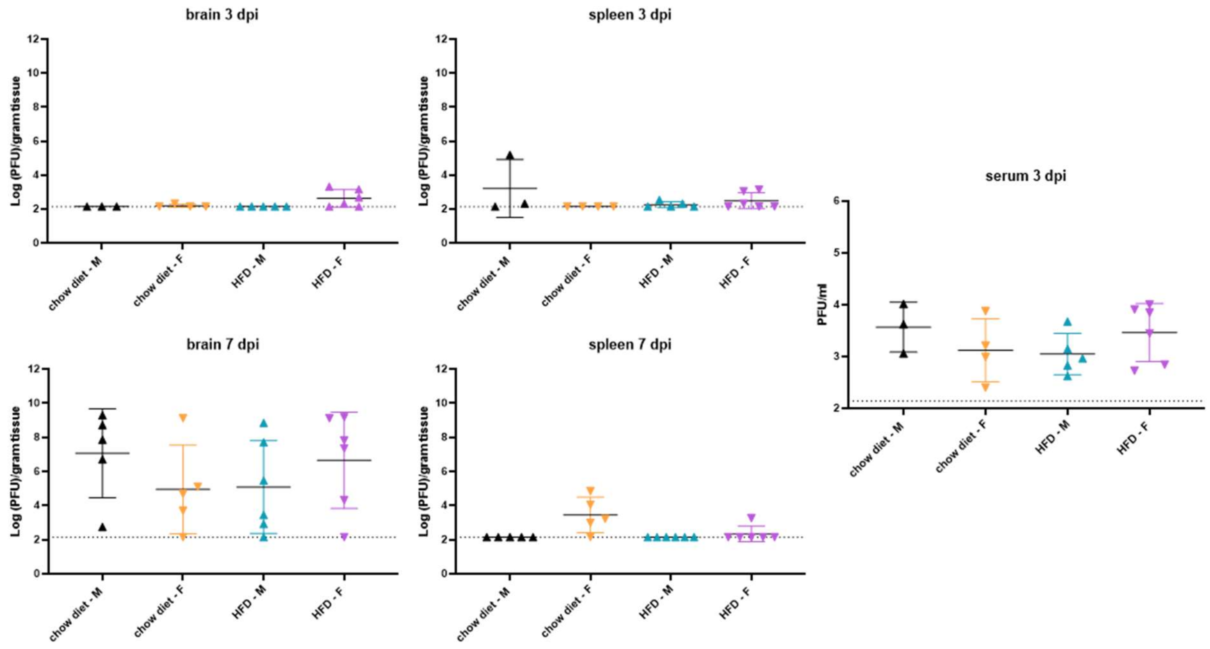

Supplementary Figure 2 | Viral titers in organs from obese and lean mice after TBEV infection. Organs and serum samples were collected at the indicated time points. Organs were homogenized and clarified by centrifugation. Serum samples and clarified organ homogenates were then subjected to plaque assays to determine viral titers.

Supplementary Table 1 | List of antibodies used for splenocyte FCM analysis

Specificity	Fluorochrome	Clone	Cat. no.	Manufacturer
MHCII	BUV395	M5/114.15.2	743876	BD Biosciences
CD19	BUV737	1D3	612782	BD Biosciences
CD38	BV421	90/CD38	562768	BD Biosciences
IgD	BV510	11-26c.2a	563110	BD Biosciences
CD95	BV605	SA367H8	152612	Biolegend
CD44	BV711	IM7	563971	BD Biosciences
IgM	FITC	RMM-1	406506	Biolegend
Sca1	RB744	D7	757296	BD Biosciences
CD22	PE	OX-97	126111	Biolegend
IgG1	PE-Dazzle594	RMG1-1	406627	Biolegend
CD138	PE/Cy7	281-2	142513	Biolegend

GL7	APC	GL7	144617	Biolegend
B220	APC/Fire810	RA3-6B2	103277	Biolegend
